# 

**Published:** 1833-09

**Authors:** 


THE LONDON
Medical and Physical Journal.

415, VOL. LXX.] SEPTEMBER 1833. [87, *New Series*.

### ADDRESS.

The Proprietors of the "London Medical and Physical Journal" having deemed it advisable to make an alteration in its size and time of appearance, think it necessary to state their reasons for the change.

When this Journal was first published, and for many years subsequently, the interval of a month between each publication was sufficiently short for the sober wishes of our medical ancestors. But, with the progress of time, new wants arose: it was found that the last blunder of a coroner, or the last squabble at a debating society, could not possibly *keep* for a month, and medical newspapers were devised, to supply this deficiency in our periodical literature. The desired object was attained; and the medical newsmonger, feasted with ever fresh intelligence, looks upon a monthly Journal with the same eye with which a London politician regards the slow Mercury or Flying Post, that satisfies the ruder taste of his rustic contemporaries. But the interval, so long to many readers, is far too short for the Editor, who, while correcting the last proof of one number, is already arranging his materials for the next. We are not desirous, therefore, to court popularity by subdividing our Journal into weekly sections, whose quadruple alliance might still form a faint imitation of our former selves. Again: the writers whose practical observations should constitute the great attraction of a work of this kind would be unwilling to entrust them to a Journal whose rapid phases would condemn their lucubrations to the destruction which, according to Southey, fugitive pamphlets both invite and deserve. We have therefore chosen the contrary alternative, and have determined that the "London Medical and Physical Journal" shall in future appear as a Quarterly Review.

It will be divided into two principal departments, namely, Reviews of English and Foreign works; and the Collectanea, or general receptacle for miscellaneous information. To these a third will occasionally, and we trust frequently, be added, namely, Original Communications; but as genius cannot be tasked, and those whose observations are most worthy of being recorded are often deficient in time or inclination to note them down, it may perhaps sometimes happen that this division may be wanting.

Our Reviews will be characterised by good temper and fairness; inclining, in doubtful cases, to mercy rather than rigid justice: yet we shall not think ourselves obliged to profess our profound respect for every careless compiler, nor shall we declare each mawkish treatise, as it drops stillborn from the press, to be an ornament to our shelves, and indispensable to the library of every medical student.

To the longer reviews of important works will be appended brief notices of those less interesting works which may not seem to demand a detailed analysis; so that the reader who looks into our pages as a mirror of current medical literature, will rarely indeed be disappointed, but will see the smaller, like the larger features, faithfully reflected.

The immense mass of practical information offered by our brother Journalists in France and Germany will be diligently analysed. Our pages will frequently be embellished by translations from these sources, which will be found under our Miscellaneous Department. Gräfe and Walther's Ophthalmological Journal, Siebold's Journal für die Geburtshelfer, (Journal for Accoucheurs,) and the well-known periodical conducted by Hufeland, will be among those to be regularly consulted.

In compiling our Collectanea, we shall aim at bringing together a large quantity of useful and readable matter, and, if we find a passage to our purpose in an old writer, we shall extract it with as little scruple as if it were in an essay just wet from the press; remembering the praise that was lavished on Oribasius of old for his diligence in studying the opinions of former physicians:

*Εἶχε γὰρ οἷα μέλισσα σοφὸν νόον, ἄλλοθεν ἄλλα*
*'Ιητρῶν προτέρων ἄνθεα δρεψάμενος.*

And now a word as to Medical Politics. We hold, with the Spartans, that in times of civil dissension it behoves every citizen to express his opinion, not shrinking from the fight, but, when necessary, taking his share of hard knocks with a good grace.

We profess, as is customary, impartiality. By this we do not mean, as is sometimes meant, that we have no opinions, but look upon right and wrong with equal indifference. We mean that, in the disputes which now agitate the medical republic, we shall regard a good argument with equal favour, whether it comes from the right or the left, believing, with Boyle, that though "testimony is like the shot of a long bow, which owes its efficacy to the force of the shooter, argument is like the shot of the cross-bow, equally forcible whether discharged by a giant or a dwarf." We shall not, therefore, in these professional skirmishes, adopt the rule of more earnest warfare, and judge the combatants by the colour of their uniform: green, blue, or brown, are alike to us. Nay, more; we shall sometimes avail ourselves of the privilege claimed from time immemorial by the umpires in matrimonial disputes, and adjudge both the contending parties to be in the wrong.

Two numbers of the Medical Quarterly Review will form a volume, which will be furnished with an ample and correct index of the matters contained in it.

The first Number will appear on the 1st of October, price five shillings; and the work will be punctually delivered to subscribers by the publisher, or any local bookseller, at 1*l.* per annum. As very few copies will be printed more than are subscribed for, it is desirable the names of subscribers should be forwarded without delay, either to their booksellers, or the publisher, J. Souter, 73, St. Paul's Church-yard.

☞ Advertisements and Bills will be charged as follows: Under six lines, 5*s.*; quarter of a page, 8*s.*; one-third, 10*s.* 6*d.*; half a page, 15*s.*; three-quarters of a page, 20*s.*; an entire page, 30*s.* Bills stitched in the Review, 30*s.*; and if above eight pages, 2*l.* 2*s.*

### OBITUARY.

DIED, on the 22d of July, at his house, in Upper Brook street, Grosvenor square, in his sixty-third year, ANTHONY MONTONNIER HAWKINS, Esq. M.D. By his family and a large circle of friends, who knew how to estimate his merits and the excellent qualities of his heart, his loss will be long and deeply deplored.

---

### APOTHECARIES' HALL.

NAMES OF GENTLEMEN to each of whom the Court of Examiners have granted Certificates:

AUGUST 1.

John Barwick, Ambleside
David Bolton, Bilston
James Culham, London
George Pyke Dowling, Bristol
Henry Inuston Gould, Rochester
Collpitts Harrison, Bishop Wearmouth
Charles Johnston, Birmingham
Thomas Prowse, Bristol
Henry Smythe, Brandon, Suffolk

AUGUST 15.

William Brown, Bradley, Staffordshire
Richard Edward Davies, Minories, London
John Gray, London
John Williams Knight
Samuel Collins Turner, Frampton on Severn

AUGUST 22.

Francis Manisty, Northumberland.

*The Subscribers to the London Medical and Physical Journal are respectfully informed, that a* TITLE-PAGE *and* INDEX *to the three last numbers of the work under the above title will be printed, and may be had of the Publisher, on the* 1st *of October, when* Part I. *of the* Medical Quarterly Review *will appear, price* 5s.

